# Supplementary material for: Modulation of Fibroblast Activity via Vitamin D3 Is Dependent on Tumor Type—Studies on Mouse Mammary Gland Cancer
Source: Cancers (Basel). 2022 Sep 21;14(19):4585. doi: 10.3390/cancers14194585 (PMC9559296; doi:10.3390/cancers14194585)
Supplement: Supplementary file 1 [file cancers-14-04585-s001.zip › cancers-1842349-supplementary.pdf]

Supplementary material for

Modulation of fibroblast activity *via* vitamin D_3 is dependent on tumor type—studies on mouse mammary gland cancers

Natalia Łabędź[1], Martyna Stachowicz-Suhs[1], Mateusz Psurski[1], Artur Anisiewicz[1], Joanna Banach[1], Aleksandra Piotrowska[2], Piotr Dzięgiel[2], Adam Maciejczyk[3,4], Rafał Matkowski[3,4], Joanna Wietrzyk[1,*]

[1]Department of Experimental Oncology, Hirszfeld Institute of Immunology and Experimental Therapy, Weigla 12, 53-114 Wroclaw, Poland
[2]Division of Histology and Embryology, Department of Human Morphology and Embryology, Faculty of Medicine, Wroclaw Medical University, Chałubińskiego 6a, 50-368 Wroclaw, Poland
[3]Department of Oncology, Wroclaw Medical University, Pl. Ludwika Hirszfelda 12, 53-413 Wroclaw, Poland
[4]Lower Silesian Oncology, Pulmonology and Hematology Center, Pl. Ludwika Hirszfelda 12, 53-413 Wroclaw, Poland

* Correspondence: joanna.wietrzyk@hirszfeld.pl; Tel. +48713709985

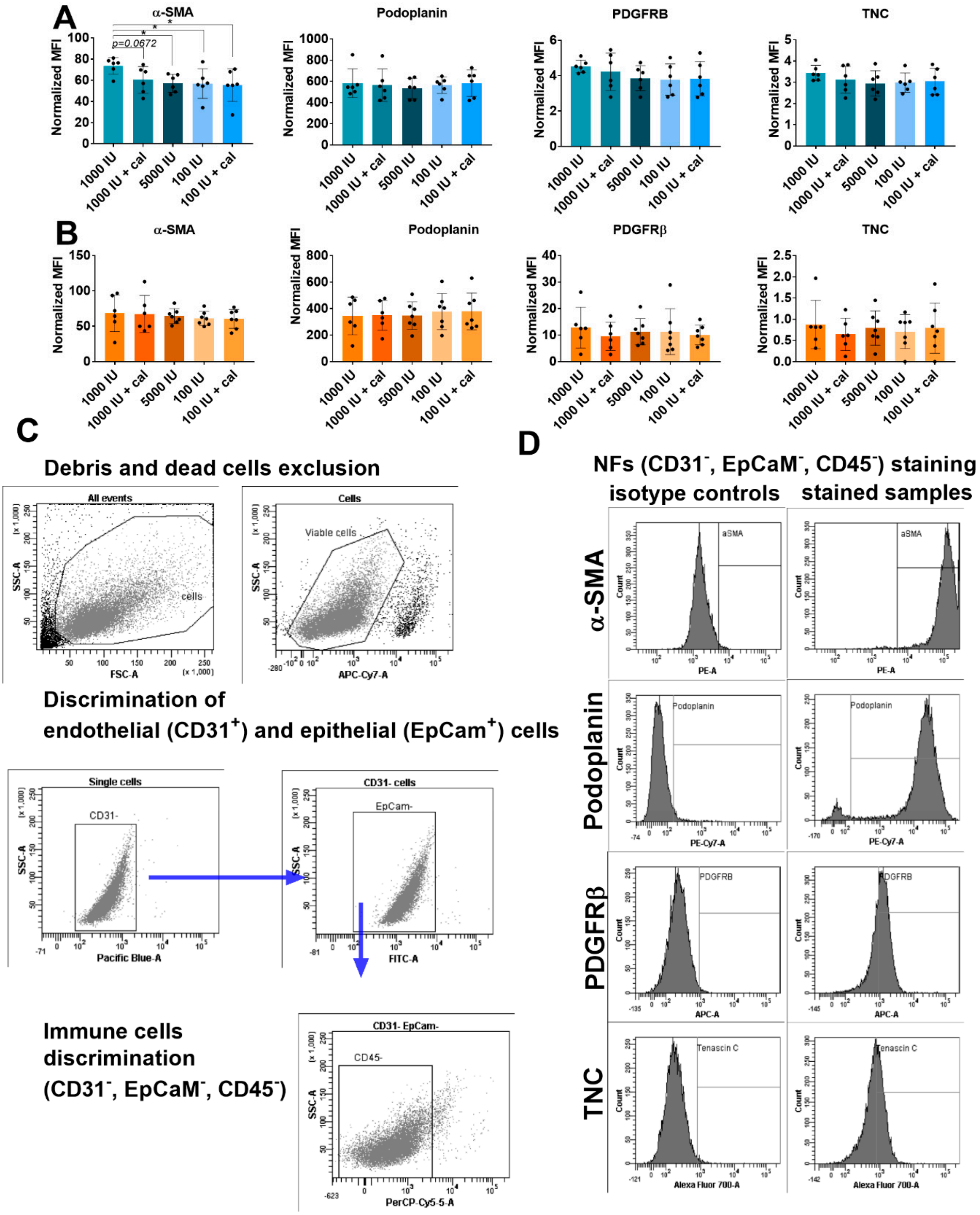

Figure S1. Flow cytometry analysis of lung NFs from healthy mice fed with different amounts of vitamin D in diets and gavaged with calcitriol. (A) BALB/c. (B) C57BL/6. Data are shown as normalized MFI calculated by dividing the MFI of the stained sample by the MFI of the isotype control. C) Steps of $CD31^{-}EpCAM^{-}CD45^{-}$ fibroblast gating. (D) Example histograms after staining of $CD31^{-}EpCAM^{-}CD45^{-}$ fibroblasts with isotype controls and appropriate antibodies. (D and E) Cells from BALB/c mice are shown as an example. Healthy mice were fed with diets containing various amounts of vitamin D for 7 weeks. Then, calcitriol gavage was started and continued thrice a week till day 23 (for C57BL/6 mice) or day 28 (for BALB/c mice). Calcitriol was administered to mice on control (1000 IU) and deficient (100 IU) diets at a dose of 1 μg/kg. During autopsy, lungs were harvested and NFs were isolated. Flow cytometry analyses were performed after short-term culture of NFs to determine the expression of

α-SMA, podoplanin, PDGFRβ, and TNC. N = 6–7. Statistical analysis: one-way analysis of variance test followed by Sidak's test for multiple comparisons. $*p < 0.05$.

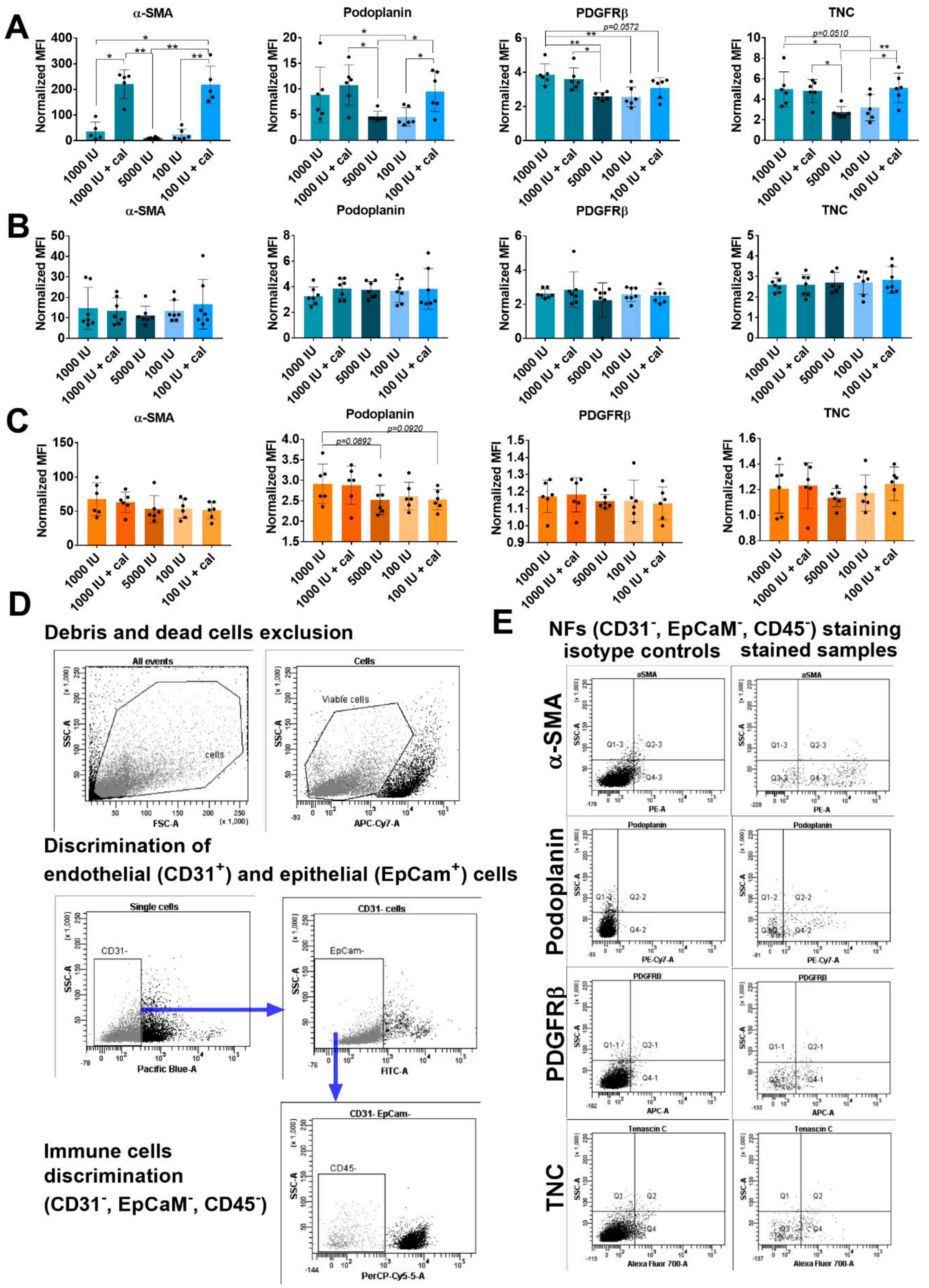

Figure S2. Flow cytometry analysis of lung NFs from mice bearing 4T1, 67NR, and E0771 tumors. (A) Mice bearing 4T1 metastatic tumors. (B) Mice bearing 67NR nonmetastatic tumors. C) Mice bearing E0771 metastatic

tumors. (A–C) Data are shown as normalized MFI calculated by dividing the MFI of the stained sample by the MFI of isotype control. (D) Steps of $CD31^{-}EpCAM^{-}CD45^{-}$ fibroblast gating. (E) Example dot-plots after staining of $CD31^{-}EpCAM^{-}CD45^{-}$ fibroblasts with isotype controls and appropriate antibodies. (D and E) Lung NFs from 4T1 tumor-bearing mice are shown as an example. Mice were fed with diets containing various amounts of vitamin D for 6 weeks. Next, on the day assigned as day 0, tumor cells were implanted orthotopically. Diets were continued for the next 7 days, and then calcitriol gavage was started and continued thrice a week till day 23 (for C57BL/6 mice) or day 28 (for BALB/c mice). Calcitriol was administered to mice on control (1000 IU) and deficient (100 IU) diets at a dose of 1 μg/kg. During autopsy, lungs were harvested and NFs were isolated. Flow cytometry analyses were performed on NFs after thawing to determine the expression of α-SMA, podoplanin, PDGFRβ, and TNC. $N = 5–7$. Statistical analysis: Kruskal–Wallis test followed by Dunn's test for multiple comparisons. $^{*}p < 0.05$; $^{**}p < 0.01$.

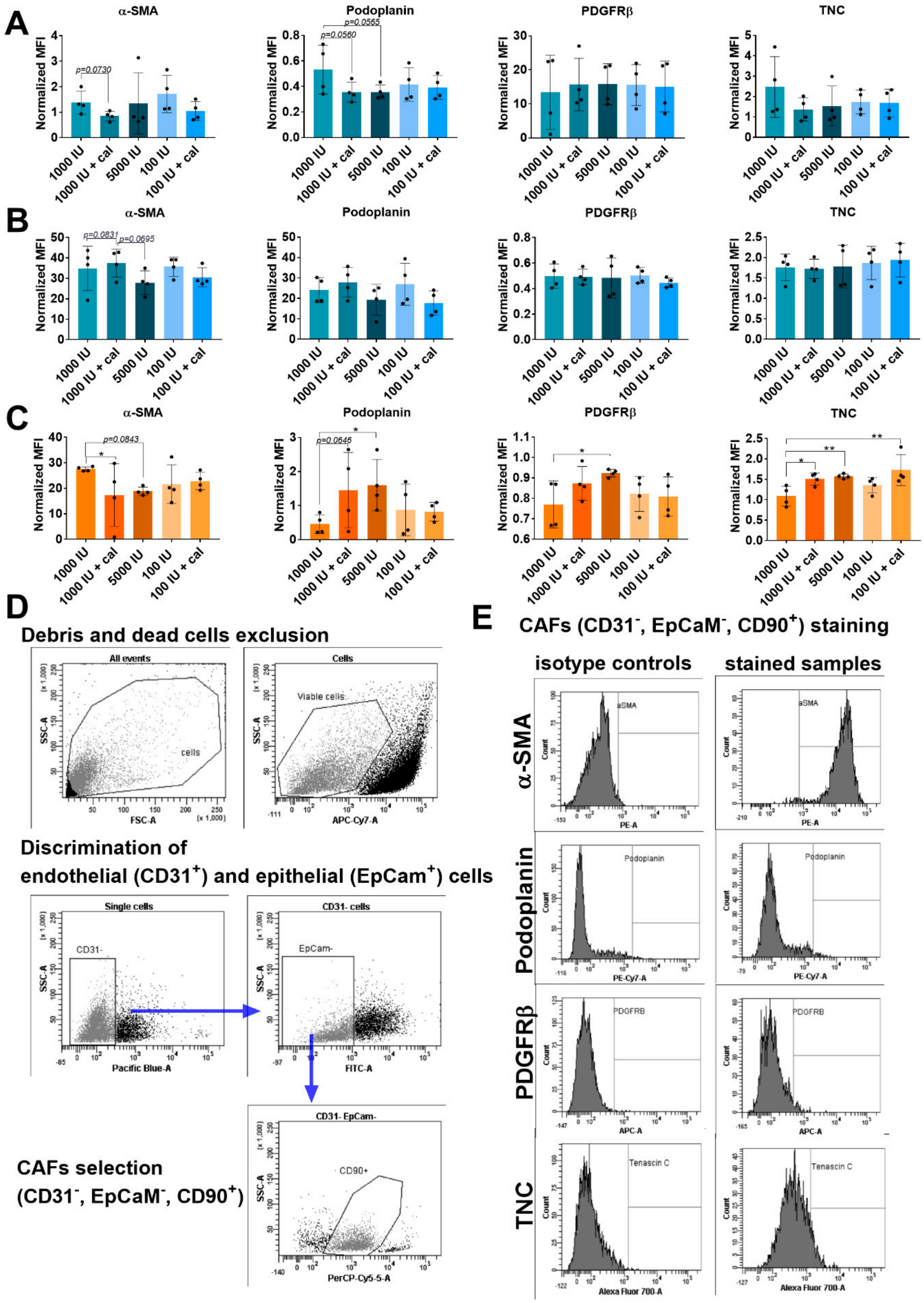

Figure S3. Flow cytometry analysis of CAFs from mice bearing 4T1, 67NR, and E0771 tumors. (A) Mice bearing 4T1 metastatic tumors. (B) Mice bearing 67NR nonmetastatic tumors. (C) Mice bearing E0771 metastatic tumors. (A–C) Data are shown as normalized MFI calculated by dividing the MFI of the stained sample by the MFI of the isotype control. (D) Steps of CD31$^-$EpCaM$^-$CD90$^+$ fibroblast gating. (E) Example histograms after staining of CD31$^-$EpCaM$^-$CD90$^+$ fibroblasts with isotype controls and appropriate antibodies. (D and E) Cells from 4T1 tumors

are shown as an example. Mice were fed with diets containing various amounts of vitamin D for 6 weeks. Next, on the day assigned as day 0, tumor cells were implanted orthotopically. Diets were continued for the next 7 days, and then calcitriol gavage was started and continued thrice a week till day 23 (for C57BL/6 mice) or day 28 (for BALB/c mice). Calcitriol was administered to mice on control (1000 IU) and deficient (100 IU) diets at a dose of 1 μg/kg. During autopsy, tumors were harvested and CAFs were isolated. Flow cytometry analyses were performed to determine the expression of α-SMA, podoplanin, PDGFRβ, and TNC. *N* = 4. Statistical analysis: Kruskal–Wallis test followed by Dunn's test for multiple comparisons. *$p < 0.05$; **$p < 0.01$.

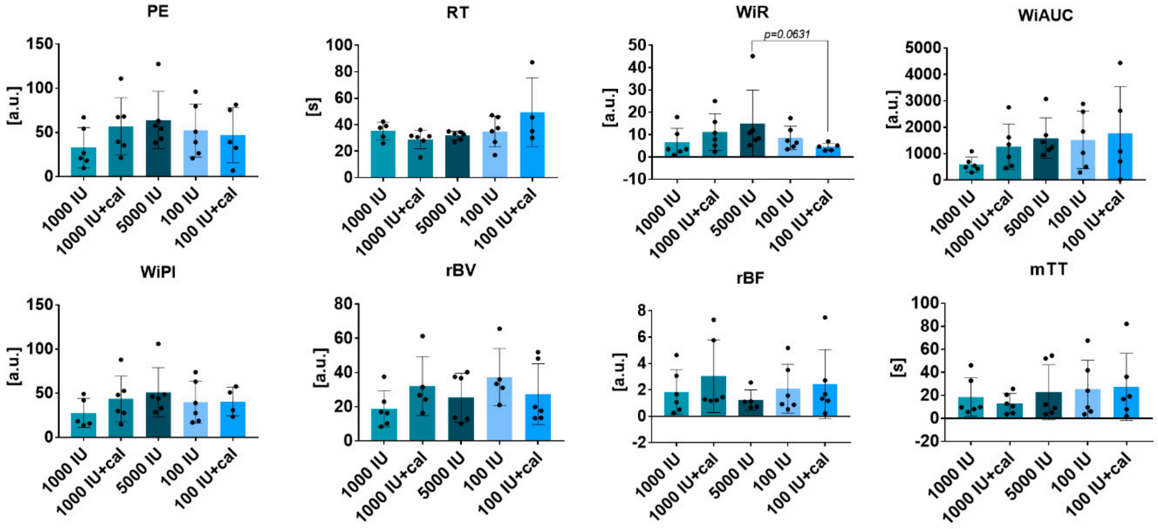

Figure S4. Blood flow in tumor tissue from 67NR tumor-bearing mice. (A) Data shown as PE representing the maximum intensity in the TIC (blood volume), TTP representing time from zero intensity to maximum intensity, RT calculated from the beginning of enhancement to PE, mTT corresponding to the center of gravity of best-fit function of echo-power (or fitted signal), WiAUC, WiR representing maximum slope between the time of onset of contrast inflow and the time of PE on the TIC, WiPI (WiAUC/RT) representing blood flow, rBV (amplitude of the plateau + offset amplitude), and rBF (rBV/mTT). Mice were fed with diets containing various amounts of vitamin D_3 for 6 weeks. Next , on the day assigned as day 0, tumor cells were implanted orthotopically. Diets were continued for the next 7 days, and then calcitriol gavage was started and continued thrice a week. Calcitriol was administered by gavage to mice on control (1000 IU) and deficient (100 IU) diets at a dose of 1 μg/kg. Blood flow parameters were measured on day 21. *N* = 4–7. Statistical analysis: analysis of variance test followed by Sidak's test for multiple comparisons. *$p < 0.05$; **$p < 0.01$.

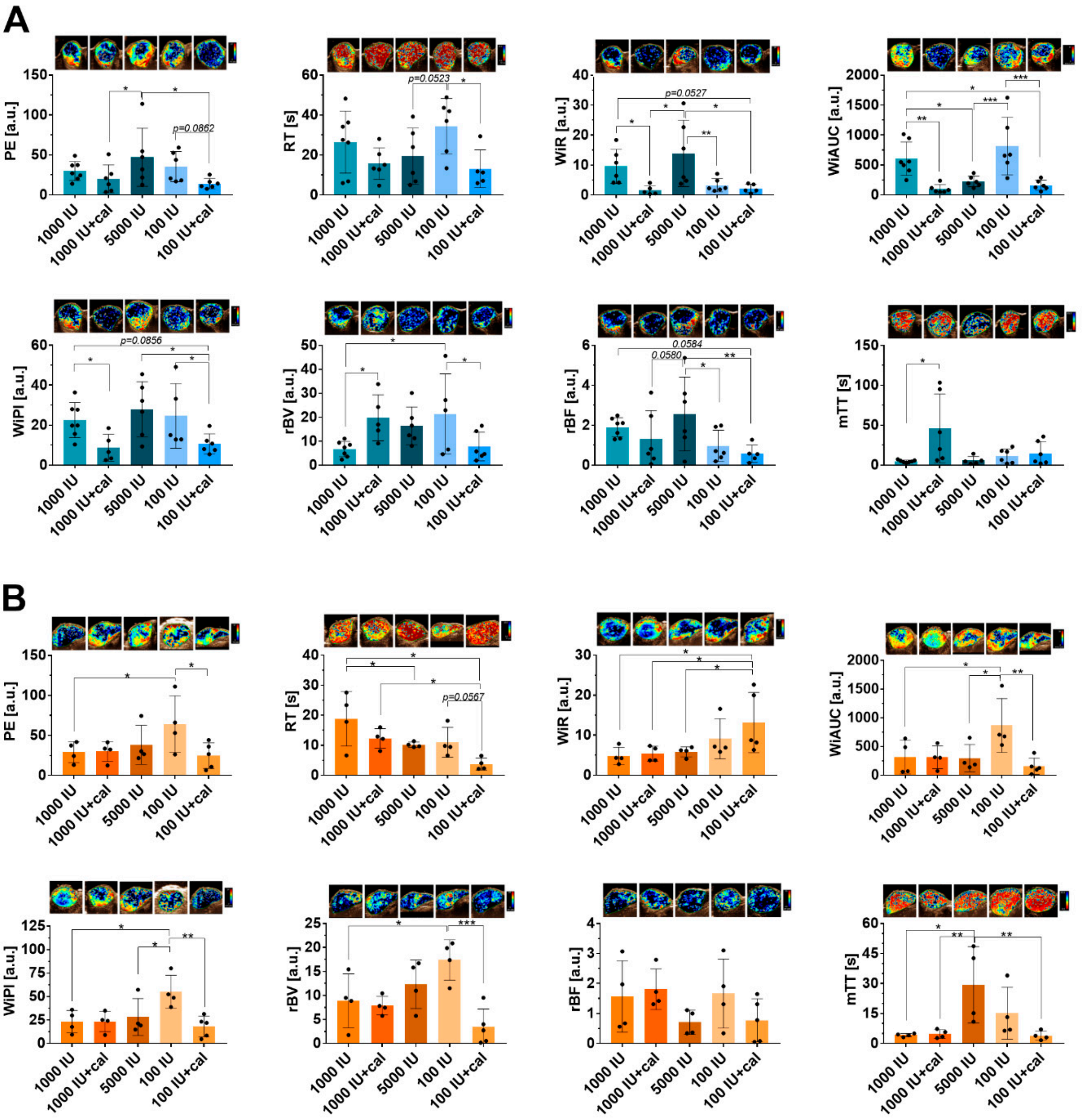

Figure S5. Blood flow in tumor tissue from 4T1 and E0771 tumor-bearing mice. (A) Mice bearing 4T1 metastatic tumors. (B) Mice bearing E0771 metastatic tumors. Data shown as peak enhancement (PE) representing the maximum intensity in the TIC (blood volume), rise time (RT) calculated from the beginning of enhancement to PE, mean transit time (mTT) corresponding to the center of gravity of best-fit function of echo-power (or fitted signal), wash-in area under the TIC curve (WiAUC), wash-in rate, maximum slope between the time of onset of contrast inflow and the time of PE on the TIC (WiR), wash-in perfusion index (WiPI = WiAUC/RT) – representing blood flow, relative blood volume (rBV = amplitude of the plateau + offset amplitude), and relative blood flow (rBF = rBV/mTT). Representative pictures of all parameters are presented. Mice were fed with diets containing various amounts of vitamin D_3 for 6 weeks. Next, on the day assigned as day 0, tumor cells were implanted orthotopically. Diets were continued for the next 7 days, and then calcitriol gavage was started and continued thrice a week. Calcitriol was administered by gavage to mice on control (1000 IU) and deficient (100 IU) diets at a dose 1 μg/kg. Blood flow parameters were measured on day 21 (A) or on day 19 (B). N = 4–7. Statistical analysis: analysis of variance test followed by Sidak's test for multiple comparisons. *$p < 0.05$; **$p < 0.01$.

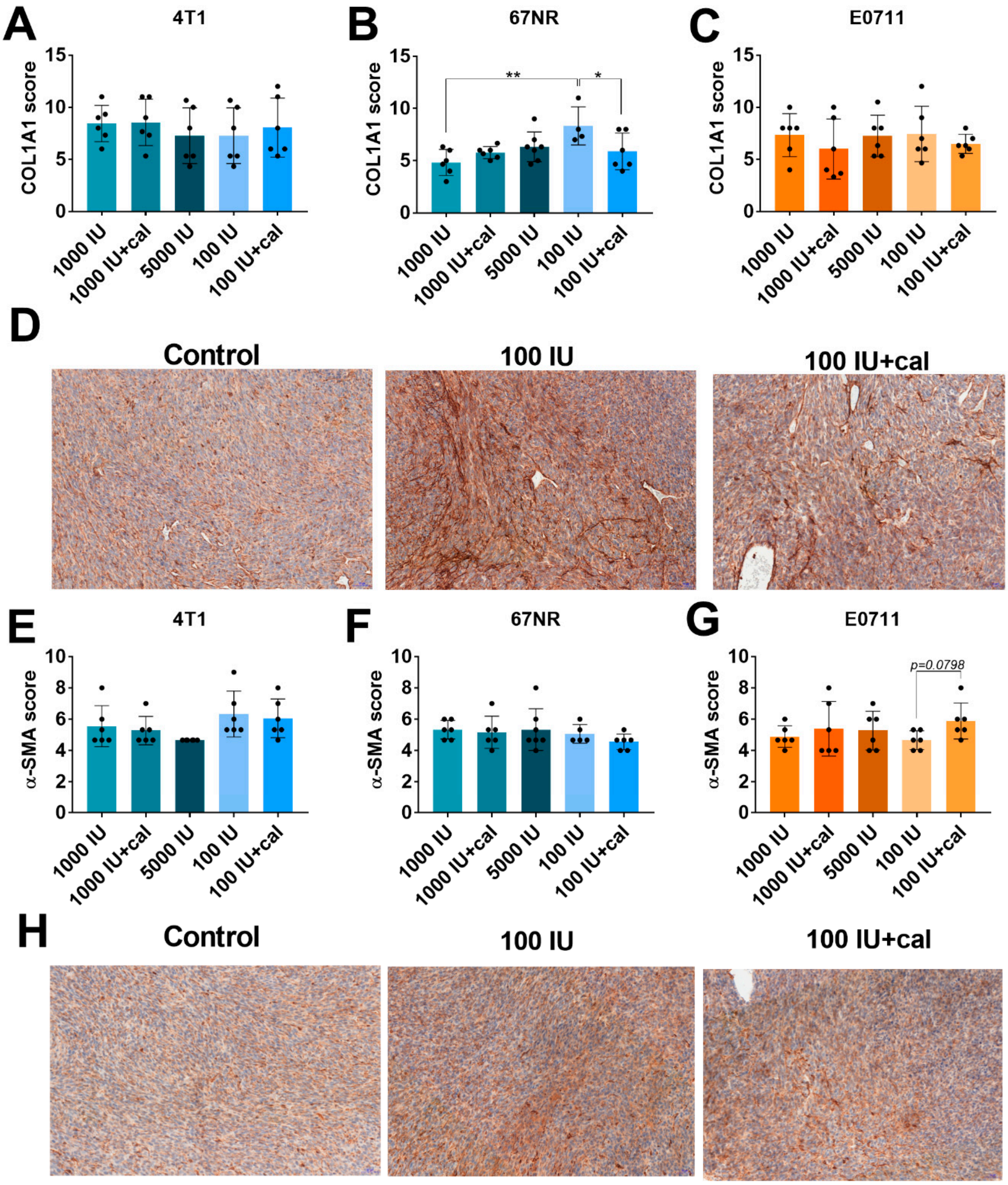

Figure S6. Tumor tissue expression of COL1A1 and α-SMA. Expression of COL1A1 in (A) 4T1, (B) 67NR, (C) E0771 tumors. (D) Example images of tumor tissue staining of 67NR tumors. Expression of α-SMA in (E) 4T1, (F) 67NR, (G) E0771 tumors. (H) Example images of tumor tissue staining of 67NR tumors. Mice were fed with diets containing various amounts of vitamin D_3 for 6 weeks. Next, on the day assigned as day 0, tumor cells were implanted orthotopically. Diets were continued for the next 7 days, and then calcitriol gavage was started and continued thrice a week. till day 23 (for C57BL/6 mice) or day 28 (for BALB/c mice). Calcitriol was administered by gavage to mice on control (1000 IU) and deficient (100 IU) diets at a dose of 1 μg/kg. During autopsy, tumors were harvested. $N = 6$. Statistical analysis: Kruskal–Wallis test followed by Dunn's test for multiple comparisons. $^{*}p < 0.05$; $^{**}p < 0.01$.

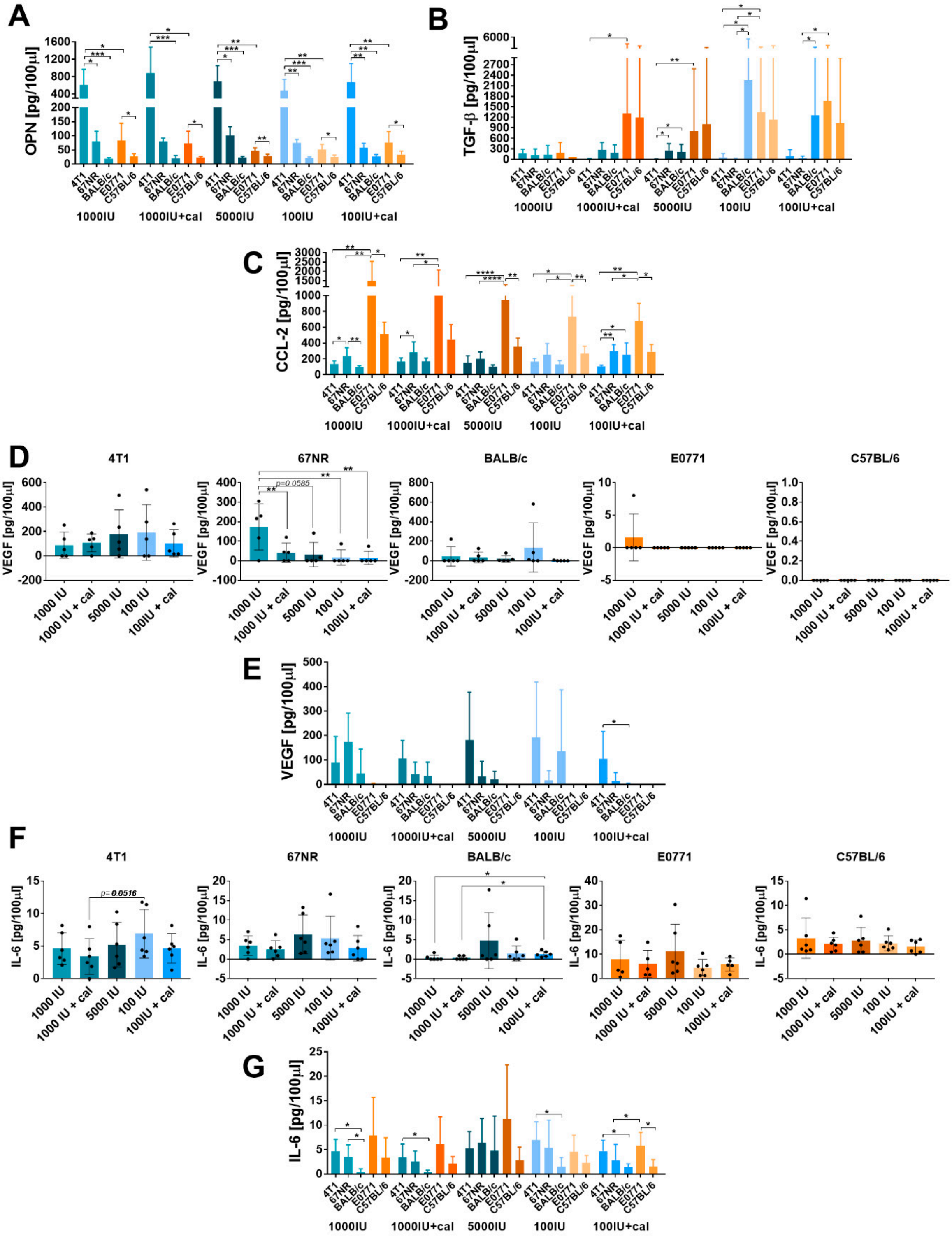

Figure S7. Plasma level of cytokines. Plasma level of (A) OPN, (B) TGF-β, (C) CCL2, (D and E) VEGF, and (F and G) IL-6 in BALB/c and C57BL/6 healthy and tumor-bearing mice. Mice were fed with diets containing various amounts of vitamin D_3 for 6 weeks. Next, on the day assigned as day 0, tumor cells were implanted orthotopically. Diets were continued for the next 7 days, and then calcitriol gavage was started and continued thrice a week till day 23 (for C57BL/6 mice) or day 28 (for BALB/c mice). Calcitriol was administered by gavage to mice on control (1000 IU) and deficient (100 IU) diets in the dose 1 µg/kg. During autopsy, blood and tumors were harvested. N = 5–7. Statistical analysis: Kruskal–Wallis test followed by Dunn's test for multiple comparisons. *$p < 0.05$; **$p < 0.01$; ***$p < 0.001$; ****$p < 0.0001$.

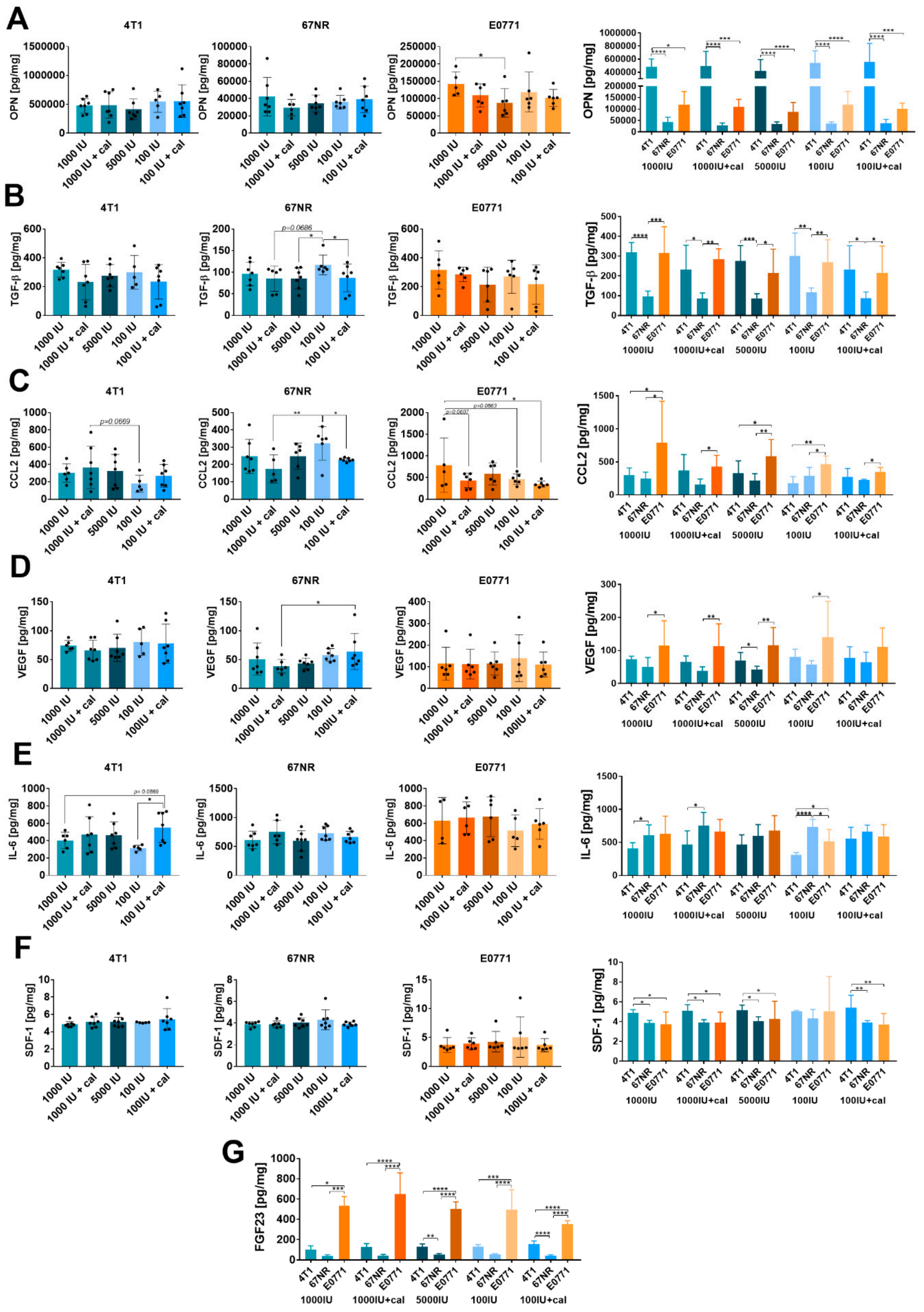

Figure S8. Tumor tissue level of cytokines. Tumor tissue level of (A) OPN, (B) TGF-β, (C) CCL2, (D) VEGF, (E) IL-6, (F) SDF-1, and (G) FGF23 in BALB/c and C57BL/6 tumor-bearing mice. Mice were fed with diets containing various amounts of vitamin D_3 for 6 weeks. Next, on the day assigned as day 0, tumor cells were implanted orthotopically. Diets were continued for the next 7 days, and then calcitriol gavage was started and continued thrice a week till day 23 (for C57BL/6 mice) or day 28 (for BALB/c mice). Calcitriol was administered by gavage to mice

on control (1000 IU) and deficient (100 IU) diets at a dose of 1 µg/kg. During autopsy, blood and tumors were harvested. N = 5–7. Statistical analysis: Kruskal–Wallis test followed by Dunn's test for multiple comparisons. *$p <$ 0.05; **p < 0.01; ***p < 0.001; ****p < 0.0001.

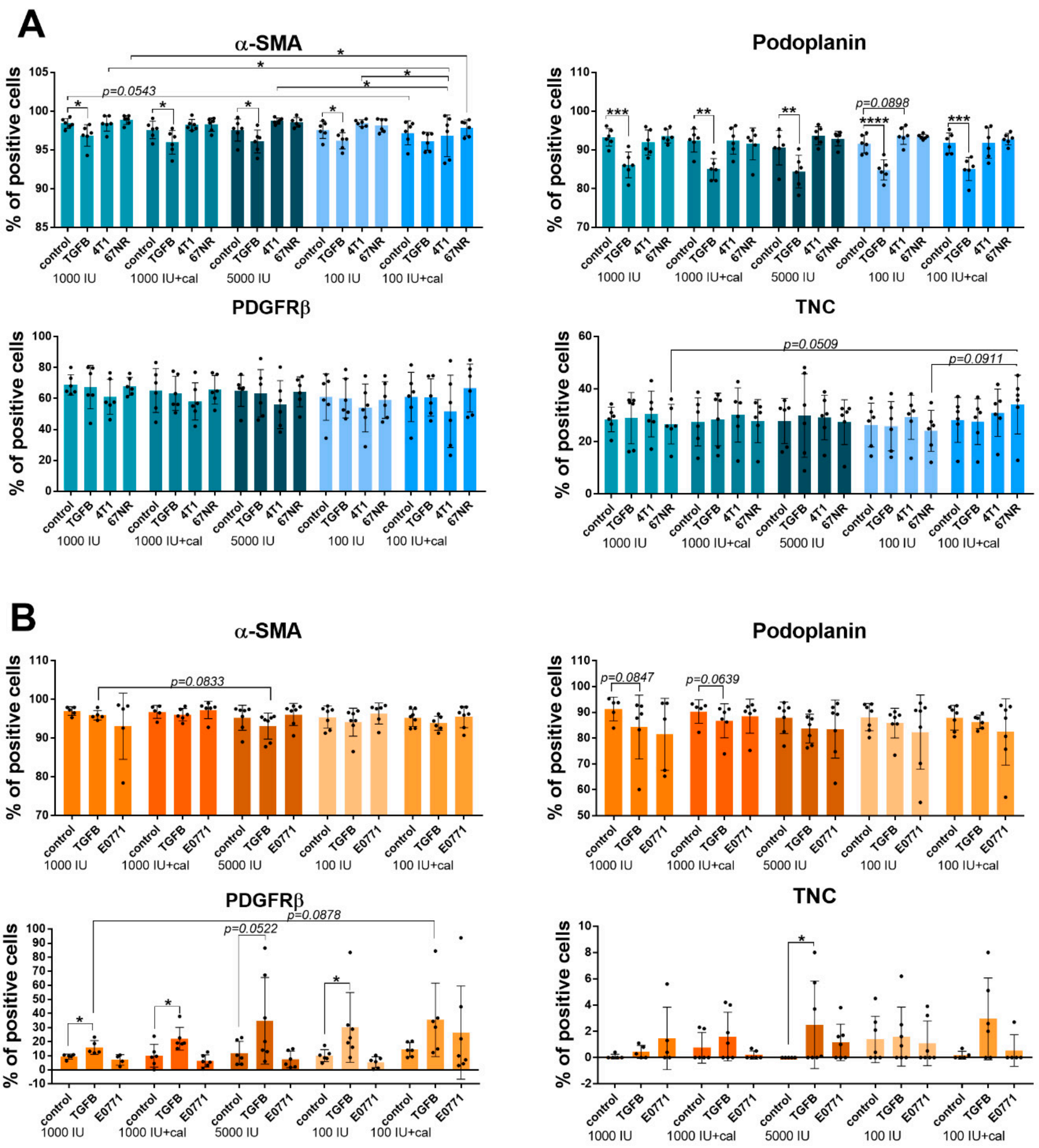

Figure S9. Phenotypic characteristics of NFs from healthy BALB/C and C57BL/6 mice treated *ex vivo* with TGF-β or CM from the culture of 4T1, 67NR (BALB/c), and E0771 (C57BL/6) cells. Data are shown as percentage of cells positive for investigated marker. Healthy mice were fed with diets containing various amounts of vitamin D for 7 weeks. Next, calcitriol gavage was started and continued TIW till day 23 (for C57BL/6 mice, B) or day 28 (for BALB/c mice, A). Calcitriol was administered to mice on control (1000 IU) and deficient (100 IU) diets at a dose of 1 µg/kg. During autopsy, lungs were harvested and NFs were isolated. Flow cytometry analyses were performed after the culture of NFs with TGF-β, 4T1, 67NR, and E0771 culture media to determine the expression of α-SMA, podoplanin, PDGFRβ, and TNC. N = 5–7. Statistical analysis: one-way analysis of variance test followed by Sidak's test for multiple comparisons. *p < 0.05; **p < 0.01; ***p < 0.001.

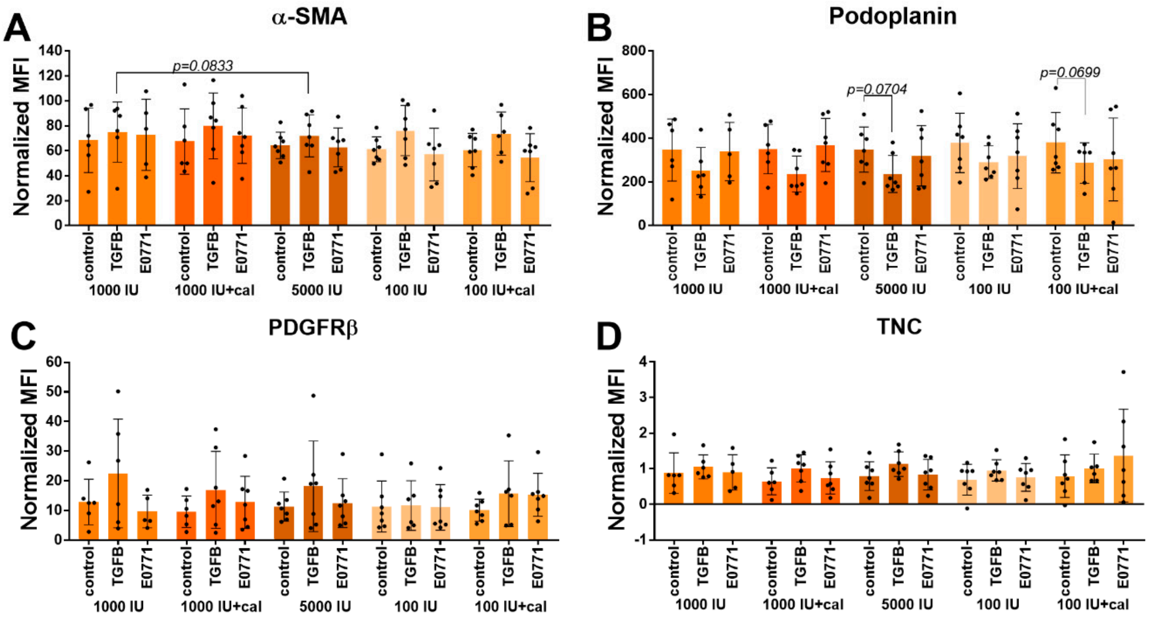

Figure S10. Phenotypic characteristics of NFs from healthy C57BL/6 mice treated *ex vivo* with TGF-β or CM from the culture of E0771 cells. Data are shown as normalized MFI calculated by dividing the MFI of the stained sample by the MFI of the isotype control. Healthy mice were fed with diets containing various amounts of vitamin D for 7 weeks. Next, calcitriol gavage was started and continued TIW till day 23. Calcitriol was administered to mice on control (1000 IU) and deficient (100 IU) diets at a dose of 1 µg/kg. During autopsy, lungs were harvested and NFs were isolated. Flow cytometry analyses were performed after the culture of NFs with TGF-β, 4T1, and 67NR culture media to determine the expression of α-SMA, podoplanin, PDGFRβ, and TNC. $N = 6–7$. Statistical analysis: one-way analysis of variance test followed by Sidak's test for multiple comparisons. *$p < 0.05$.
